# Anti-*Candida* activity of antimicrobial impregnated central venous catheters

**DOI:** 10.1186/s13756-017-0269-x

**Published:** 2017-11-03

**Authors:** L. Cobrado, A. Silva-Dias, M. M. Azevedo, A. Rodrigues

**Affiliations:** 1 Division of Microbiology, Department of Pathology, Faculty of Medicine, University of Porto, Alameda Prof. Hernâni Monteiro, 4200 Porto, Portugal; 2 Burn Unit, Department of Plastic and Reconstructive Surgery, Centro Hospitalar São João, Porto, Portugal; 3 CINTESIS, Center for Health Technology and Services Research, Faculty of Medicine, University of Porto, Porto, Portugal

**Keywords:** Healthcare-associated infections, Antimicrobial central venous catheters, Candidemia

## Abstract

**Background:**

Whenever the rate of central line-associated bloodstream infections (CLABSIs) remains high even after the implementation of preventive strategies, the use of chlorhexidine/silver sulfadiazine (CSS) or minocycline/rifampin (MR)-impregnated central venous catheters (CVCs) is currently recommended. Nevertheless, the efficacy of such CVCs against *Candida albicans* and other emerging non-*albicans* spp. has been insufficiently studied. This study aims to compare the activity of CSS and MR-impregnated CVCs against the yeasts most frequently isolated from CLABSIs.

**Methods:**

For biofilm formation assays, type strains and clinical isolates of *C. albicans*, *C. glabrata* and *C. parapsilosis* sensu stricto were used. Segments of standard polyurethane, MR and second-generation CSS-CVCs were tested. The biofilm metabolic activity was measured by a semi-quantitative XTT reduction assay.

**Results:**

CSS catheter segments significantly reduced the biofilm metabolic activity by all tested *Candida* spp., with inhibition ranging from 60% to 100%. The MR catheter segments promoted *C. albicans* and *C. parapsilosis* biofilm formation and exhibited an inconspicuous effect against *C. glabrata*.

**Conclusions:**

Among the recommended antimicrobial CVCs, CSS-CVCs proved to be superior in the inhibition of biofilm formation by the most frequent yeasts causing CLABSIs. Data from this in vitro study may suggest that patients at high risk for invasive candidosis could benefit from the use of CSS-CVCs.

## Background

Although there are some signs of a possible decline in the incidence of central line-associated bloodstream infections (CLABSIs) [1], it certainly remains a major medical concern for which further preventive measures can be taken to minimize its impact upon morbidity, increased length of hospital stay and financial resources expended. A 46% decrease in the incidence has occurred in US hospitals from 2008 to 2013, even though an estimated 30,100 CLABSIs still occur each year [2]. As reported by the European Centre for Disease Prevention and Control, bloodstream infections may be catheter-related in 43.3% of cases, with a mean device-adjusted rate in patients staying in the Intensive Care Unit (ICU) for more than 2 days of 3.0 CLABSI episodes per 1000 CVC-days. Among isolated microorganims in ICU-acquired bloodstream infections from participant EU countries, *Candida* spp. represented a total of 8.2% [3].

Globally, *Candida* spp. are the fourth frequent causative pathogen of CLABSIs [4] and contribute to 12% of all CLABSIs [5], with an attributable mortality of 38% [6]. Through the colonization of the skin, manipulation of the catheter hub, contamination of infusates or from a distant focus of infection, *Candida* cells may adhere to the CVC surface and produce extracellular polymers, providing a structural matrix that will further facilitate adhesion [7]. In such biofilm, *Candida* cells display an increased resistance to antifungal agents and, from such strategic anchorage point, yeasts may disperse into the bloodstream, leading to serious infection [7–9].

Despite being a heterogeneous group of organisms, most of the invasive infections are caused by *C. albicans*, *C. parapsilosis* and *C. glabrata* [10, 11]. Risk factors for invasive candidosis include the presence of a CVC, prolonged ICU stay, use of broad-spectrum antibiotics, previous gastrointestinal surgery, neutropenia, diabetes mellitus, burns and administration of total parenteral nutrition [12, 13]. Moreover, a trend towards infections by non-*albicans* spp. has been noticed in the past years [14–16]. This change in epidemiology can be associated with severe immunosuppression or illness, exposure to broad-spectrum antibiotics, prematurity or older age [14].

Recommendations for CLABSI prevention issued by the US Centers for Disease Control and Prevention (CDC) target, for example, the education of healthcare personnel, hand hygiene and aseptic techniques for insertion and care of central venous catheters (CVCs) [17]. Even though, whenever the rate of infection remains high even after successful implementation of a comprehensive strategy to reduce CLABSIs, the use of chlorhexidine/silver sulfadiazine (CSS) or minocycline/rifampin (MR)-impregnated CVCs is currently recommended [17]. According to the respective manufacturer’s technology sheet, second-generation CSS-CVCs (coated on both the external and internal surfaces) should exhibit antimicrobial activity against *Klebsiella pneumoniae*, *Escherichia coli*, *Pseudomonas aeruginosa*, *Staphylococcus aureus*, *S. epidermidis* and *Candida albicans* [18] and MR-CVCs are expected to display a broad-spectrum protection against Gram-positive, Gram-negative and fungal infections [19]. In spite of this wide antimicrobial spectrum, the efficacy of impregnated CVCs against yeasts, particularly non-*albicans* species*,* has not been fully addressed. Moreover, if effectiveness of MR-CVCs against *Candida* has been previously demonstrated [20, 21], some authors have reported more recently that MR-CVCs may be prone to colonization by this yeast [22–24].

Therefore, the aim of this manuscript is to compare and clarify the efficacy of the recommended CSS and MR-impregnated CVCs against the most frequent yeasts isolated from CLABSIs, in order to help clinicians to make the best option for patients at high risk of invasive *Candida* infection, whenever other preventive strategies have failed.

## Methods

### Strains

Type strains belonging to the American Type Culture Collection (ATCC) or to the Centraalbureau voor Schimmelcultures (CBS) were used, along with clinical isolates of *C. albicans*, *C. glabrata* and *C. parapsilosis* sensu stricto previously collected from patients admitted at a tertiary university hospital and identified using the Vitek 2 system (bioMérieux, France) (Table 1).

**Table 1:** Microbial strains used: distribution by species and provenance

Strain	Isolate	Site of isolation or Source
ATCC 90028	*C. albicans*	American Type Culture Collection
CA075	*C. albicans*	Central venous catheter
ATCC 22019	*C. parapsilosis*	American Type Culture Collection
CP007	*C. parapsilosis*	Central venous catheter
CBS138	*C. glabrata*	CBS Collection
CG044	*C. glabrata*	Central venous catheter

Yeast strains were kept frozen in yeast peptone dextrose medium (YPD) (Formedium, Hunstanton, England) supplemented with 40% glycerol at −70 °C until testing. For each experiment, microorganisms were subcultured twice on Sabouraud agar (Liofilchem, Italy), at 35 °C, during 24 h to assess the purity of the culture and its viability.

### Biofilm formation

#### Medium and growth conditions

*Candida* strains were grown overnight in Sabouraud broth at 37 °C and 180 rpm. Afterwards, yeast cells were harvested by centrifugation, washed with PBS and standardized to 1 × 10^6^ cells/mL in Roswell Park Memorial Institute (RPMI) 1640 medium supplemented with L-glutamine and buffered with MOPS acid (Sigma-Aldrich).

#### Biofilm formation assays

Three different central venous catheters were used as substrate for the biofilm formation assays: standard uncoated polyurethane double-lumen central venous catheters (Arrow® International, Inc. Reading, PA, USA), minocycline-rifampin impregnated central venous catheters (Cook® Medical, Bloomington, Inc., USA) and second-generation chlorhexidine/silver sulfadiazine-impregnated central venous catheters (Arrowgard Blue® Plus, Arrow® International, Inc., Reading, PA, USA). For biofilm assays, 1-cm segments of each catheter were used. For each experimental condition, 3 catheter segments were used and experiments were performed twice, in different days.

To evaluate biofilm formation in the catheters, 2 mL of standardized yeast suspensions (1 × 10^6^ cells/mL) [25] were added to glass vials containing one segment of each catheter and allowed to form biofilm for 24, 48 and 72 h, at 37 °C, with agitation (180 rpm). At 24 and 48 h time points, the catheters were washed with PBS and placed in a new tube with 2 mL of fresh RPMI. Catheters incubated only with RPMI were used as negative controls. For each time point, catheters were washed with PBS and the biofilm metabolic activity was quantified using the semi-quantitative XTT reduction assay [25]. Briefly, 2 ml of a XTT/menadione solution (0.5 g/L and 1 μM respectively) were added to each catheter and incubated in the dark for 3 h at 37 °C. Then the supernatant absorbances were read in a spectrophotometer at 490 nm.

### Statistical analysis

Biofilm formation, in different time points, was evaluated with One-Way ANOVA (with the Bonferroni correction. Statistical significance was considered as a *p* value smaller than 0.05. All statistical analysis was performed using the SPSS software (v. 20.0).

## Results

### Biofilm formation on standard polyurethane central venous catheters

**Fig. 1:**
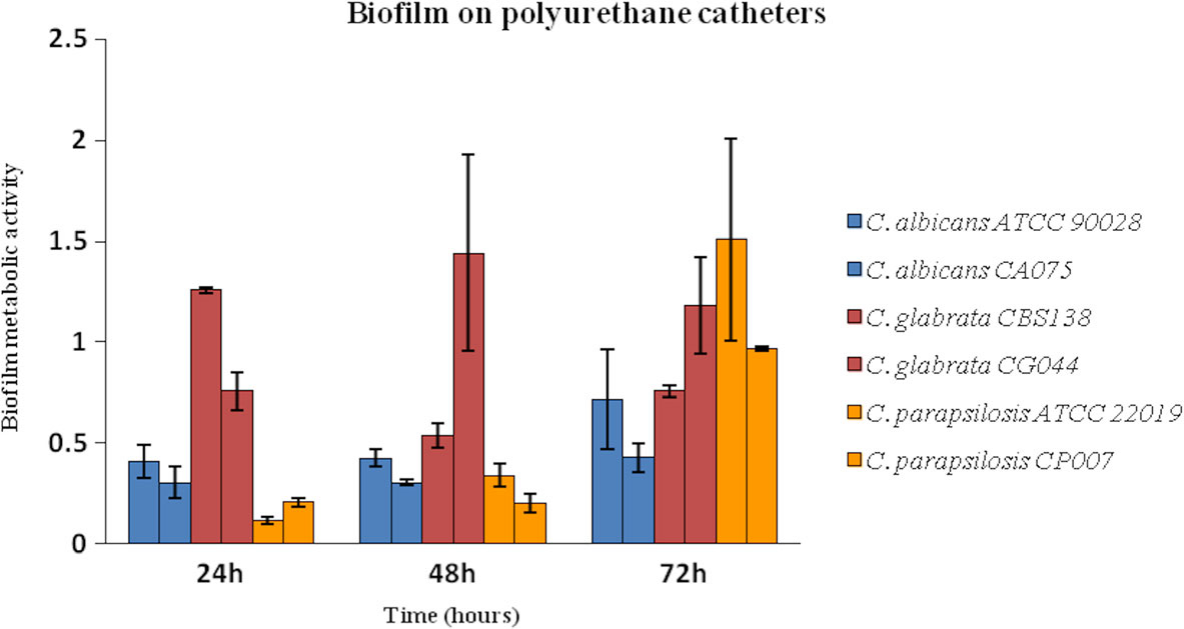
Biofilm formation by different *Candida* species on standard uncoated polyurethane catheters at 24, 48 and 72 h. Biofilms were quantified colorimetrically by XTT assay, which measures biofilm metabolic activity. Error bars represent the standard deviation among the results for different isolates. Each isolate was tested for its ability to form biofilm at least 6 times

*C. glabrata* was the species that showed higher values of biofilm metabolic activity at 24 h and 48 h time periods. At these time periods, *C. albicans* and *C. parapsilosis* exhibited similar values of biofilm metabolic activity (Fig. 1).

At the 72 h time point, all the species had produced biofilm with higher metabolic activity when compared with the 24 and 48 h periods. *C. glabrata* and *C. parapsilosis* exhibited the higher values of biofilm formation at this period.

Absorbance values for negative controls showed the absence of background or contamination (values equal to zero).

### Effect of antimicrobial catheters in biofilm formation

**Fig. 2:**
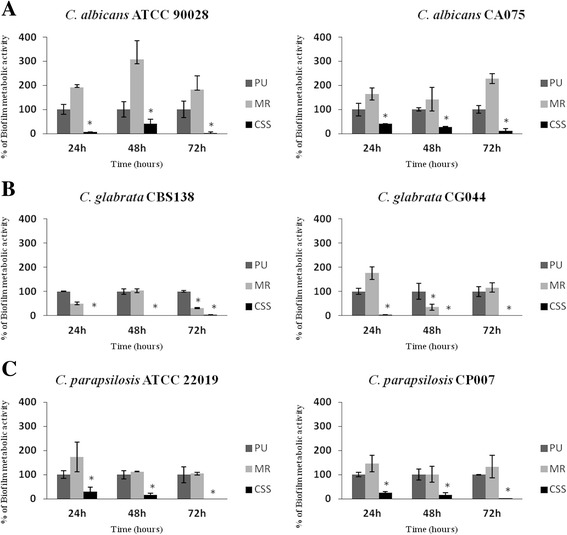
In vitro biofilm formation on central venous catheters. Distinct *Candida* species were allowed to form biofilm for 24, 48 and 72 h in segments of three different CVCs: standard uncoated polyurethane CVC (PU), minocycline-rifampin impregnated CVC (MR) and second-generation chlorhexidine/silver sulfadiazine-impregnated (CSS). Graphics show the results for the two isolates of *C. albicans* (**a**), *C. glabrata* (**b**) and *C. parapsilosis* (**c**). Error bars represent the standard deviation among the results for different isolates. Each isolate was tested for its ability to form biofilm at least 6 times. * *P* < 0.05 of coated catheters when compared with PU at each time point

In general, the CSS catheter segments were the most efficient in preventing biofilm formation by all *Candida* species (Fig. 2a). The MR catheter segments were not able to prevent biofilm formation by all these species, promoting its development in some cases.

Regarding *C. albicans*, the CSS catheter segments were effective in biofilm inhibition for all strains at all the studied time points. The inhibition ranged from 60% to 93%. The MR catheter segments promoted *C. albicans* biofilm formation, at all tested conditions (Fig. 2a).

Concerning *C. glabrata*, the species that produced more biofilm, CSS catheter segments were also efficient in biofilm inhibition, for all strains and time points, ranging from 97 to 100% of inhibition (Fig. 2b). The MR catheters showed a slight reduction in the strain CBS 138 at 24 and 72 h time points and for strain CG044 at 48 h time point. However, reduction was never inferior to 50% of biofilm metabolic activity (Fig. 2b).

*C. parapsilosis* biofilm formation was also inhibited in a large extent by the CSS catheter segments (70 to 100% of biofilm inhibition), at all studied time points (Fig. 2c). The MR catheters were unable to prevent biofilm formation in this species, at all tested conditions (Fig. 2c).

Absorbance values for negative controls showed the absence of background or contamination (values equal to zero).

## Discussion

CLABSIs remain a global medical concern and further work on preventive measures must definitely be pursued in order to reduce its incidence. Hence, when CLABSI rate is high, the CDC strongly recommends the implementation of a strategy that should include the education of healthcare personnel responsible for catheter insertion and maintenance, the use of maximal sterile barrier precautions and skin antisepsis using >0.5% chlorhexidine preparation with alcohol during CVC insertion. Whenever after successful implementation of such comprehensive strategy the CLABSI rate is not decreasing, the CDC equally recommends the use of CSS or MR-impregnated CVCs in patients whose catheter is expected to remain in place >5 days, since such catheters can decrease the risk for CLABSI and thus result in lower hospital final costs [17].

Compared to standard non-coated catheters, first-generation CSS-CVCs (coated only on the external luminal surface) did reduce the risk for CLABSI [26, 27]. Regarding the more recently available second-generation CVCs, a significant reduction in catheter colonization has been demonstrated, but a clear difference in CLABSI rate was not found due to low power of the randomized studies [28–30]. In the present biofilm formation experiment, CSS- impregnated CVCs effectively reduced biofilm formation by the most frequent yeast species causing CLABSIs, with inhibitions that ranged from 60% to 100%. These strong inhibitory results certainly lack validation by clinical studies. The use of CSS-CVCs is not without concerns of hypersensitive reactions [31–33]. Even though, the CDC reports that the use of CSS-CVCs might be cost effective in ICU, burn and neutropenic patients and in other patient populations with rates of infection exceeding 3.3 per 1000 catheter days [34]. From a novel perspective, data from our experiment may further suggest that patients at high risk for invasive candidosis could benefit from the use of CSS-impregnated CVCs.

MR-CVCs (coated on both the internal and external luminal surfaces) were associated with lower rates of CLABSI when compared with first-generation CSS-CVCs [35]. Since then, scarcer and less powered studies have been made comparing MR-CVCs with second-generation CSS-CVCs. Moreover, some controversy may still exist regarding the activity of MR-CVCs against yeasts: some authors have found a broad-spectrum activity against several bacteria and *C. albicans* [20, 36] and the technology sheet of the manufacturer of MR-CVCs reports activity against fungal infections. However, in our in vitro experiment, the MR-CVC segments were not only unable to prevent biofilm formation by *C. albicans* and *C. parapsilosis* strains, but also promoted biofilm formation by both yeasts, most markedly in the case of *C. albicans*. Regarding *C. glabrata,* the MR-CVC segments showed an inconspicuous effect, with a slight reduction in biofilm formation only at some time points. Using another in vitro model and considering *C. albicans*, Gaonkar et al. found similar results with increased adherence of the yeast to MR-CVCs compared to control CVCs [37]. Furthermore, on the meta-analysis of randomized controlled trials performed by Falagas et al., evidence was found that MR-impregnated catheters were prone to colonization with *Candida* [38]. Nevertheless, clinical trials have not found an increased incidence of CLABSIs due to *Candida* spp., even though the analyzed studies were underpowered to detect such difference. In vivo increased colonization of MR-CVCs by *Candida* spp. may result from the exposure of both surfaces of the catheter to a higher and continuous bacterial challenge from the hub and the insertion site [24]. Issues concerning the emergence of resistant pathogens with the use of MR-CVCs have been raised [39, 40], but still remain undocumented in the clinical setting [17].

In our study, a validated XTT reduction assay was used to evaluate the susceptibility of *Candida* biofilms when exposed to CSS and MR-impregnated CVCs [41]. One limitation of this assay that quantifies biofilm by colorimetric analysis is that it may not always correlate metabolic activities between different species [42]. However, this was not the case of our experiment, since the activity of CSS and MR-CVCs was compared using the same strains. Furthermore, in accordance with our results and using a different method (assessment of colony forming units with a modified Kirby-Bauer technique), Hanna et al. found not only that CSS-CVCs were superior against *C. albicans* and *C. parapsilosis* adherence, but also that the use of MR-CVC increased the colonization by *Candida* spp. [43].

In a meta-analysis conducted by Novikov et al. in hospitalized patients comparing the incidence of specific bacterial and fungal species colonizing antimicrobial CVCs and standard CVCs, it was found that antimicrobial CVCs in clinical use may become colonized with distinct microorganisms probably in relation to their antimicrobial spectrum of activity. In the particular case of *Candida* spp., the proportion of colonized MR-CVCs was greater than that of colonized standard CVCs [44]. Hence, antimicrobial CVCs may be advantageous over standard CVCs for specific microbial pathogens. As our experiment demonstrated, CSS-CVC exhibits an excellent activity against biofilm formation by the yeasts responsible for the majority of invasive candidosis; thus, patients at increased risk could well benefit from the use of such CSS-impregnated CVCs.

Instead of using clinical risk factors to tailor the selection of the best CVC for a given patient, alternatives are being studied targeting a wider antimicrobial spectrum per catheter. This is the case of the catheter impregnated with chlorhexidine (CHX) and MR, as proposed by Raad et al.: CHX-MR CVC may be more effective in completely inhibiting the biofilm formation by resistant bacteria and fungi, with prolonged antimicrobial activity [45]. Nevertheless, the implementation of this CVC would certainly benefit from further clinical experience and validation. Current CDC recommendations still contemplate nothing but two options: CSS and MR-impregnated CVCs. Another CVC impregnated with rifampicin-miconazole has been associated with a significantly lower risk of catheter colonization and CLABSIs compared to standard catheters in a prospective clinical trial in two university hospitals [46] and with a statistically significant reduction in the incidence of catheter-related bacteremia in patients with short-term catheter use at the central jugular and femoral sites in an ICU setting [47], but, again, further clinical validation and powered multicenter randomized studies are needed.

## Conclusions

In this biofilm formation experiment, CSS-CVCs had a superior anti-*Candida* activity comparing to MR-CVCs. CSS-CVCs exhibited a strong antibiofilm activity against *C. albicans* and, moreover, against the emerging *C. glabrata* and *C. parapsilosis.* This study further documented not only the lack of antibiofilm activity of MR-CVCs against the tested yeasts, but also the promotion of biofilm formation in in vitro conditions, particularly of *C. albicans*. Therefore, following current CDC recommendations to use antimicrobial impregnated CVCs whenever CLABSI rate remains high and regarding patients at serious risk of invasive *Candida* infection, the use of CSS-CVCs may be suggested.
